# The Burden of Oral Disease among Perinatally HIV-Infected and HIV-Exposed Uninfected Youth

**DOI:** 10.1371/journal.pone.0156459

**Published:** 2016-06-14

**Authors:** Anna-Barbara Moscicki, Tzy-Jyun Yao, Mark I. Ryder, Jonathan S. Russell, Stephen S. Dominy, Kunjal Patel, Matt McKenna, Russell B. Van Dyke, George R. Seage, Rohan Hazra, Shiboski

**Affiliations:** 1 Department of Pediatrics, Division of Adolescent Medicine, University of California, Los Angeles, Los Angeles, California, United States of America; 2 Center for Biostatistics in AIDS Research (CBAR), Harvard T.H. Chan School of Public Health, Boston, Massachusetts, United States of America; 3 Department of Orofacial Sciences, School of Dentistry, University of California, San Francisco, San Francisco, California, United States of America; 4 Department of Psychiatry, School of Medicine, University of California, San Francisco, San Francisco, California, United States of America; 5 Department of Epidemiology, Center for Biostatistics in AIDS Research (CBAR), Harvard T.H. Chan School of Public Health, Boston, Massachusetts, United States of America; 6 Department of Pediatrics, School of Medicine, Tulane University, New Orleans, Louisiana, United States of America; 7 Department of Epidemiology, Harvard T.H. Chan School of Public Health, Boston, Massachusetts, United States of America; 8 Maternal and Pediatric Infectious Disease Branch at the Eunice Kennedy Shriver National Institute of Child Health and Human Development (NICHD), National Institutes of Health (NIH), Bethesda, Maryland, United States of America; UCL Institute of Child Health, University College London, UNITED KINGDOM

## Abstract

**Objective:**

To compare oral health parameters in perinatally HIV-infected (PHIV) and perinatally HIV-exposed but uninfected youth (PHEU).

**Methods:**

In a cross-sectional substudy within the Pediatric HIV/AIDS Cohort Study, participants were examined for number of decayed teeth (DT), Decayed, Missing, and Filled Teeth (DMFT), oral mucosal disease, and periodontal disease (PD). Covariates for oral health parameters were examined using zero-inflated negative binomial regression and ordinal logistic regression models.

**Results:**

Eleven sites enrolled 209 PHIV and 126 PHEU. Higher DT scores were observed in participants who were PHIV [Adjusted Mean Ratio (aMR) = 1.7 (95% CI 1.2–2.5)], female [aMR = 1.4 (1.0–1.9)], had no source of regular dental care [aMR = 2.3 (1.5–3.4)], and had a high frequency of meals/snacks [≥5 /day vs 0–3, aMR = 1.9 (1.1–3.1)] and juice/soda [≥5 /day vs 0–3, aMR = 1.6 (1.1–2.4)]. Higher DMFT scores were observed in participants who were older [≥19, aMR = 1.9 (1.2–2.9)], had biological parent as caregiver [aMR = 1.2 (1.0–1.3)], had a high frequency of juice/soda [≥5 /day vs 0–3, aMR = 1.4 (1.1–1.7)] and a low saliva flow rate [mL/min, aMR = 0.8 per unit higher (0.6–1.0)]. Eighty percent had PD; no differences were seen by HIV status using the patient-based classifications of health, gingivitis or mild, moderate, or severe periodontitis. No associations were observed of CD4 count and viral load with oral health outcomes after adjustment.

**Conclusions:**

Oral health was poor in PHIV and PHEU youth. This was dismaying since most HIV infected children in the U.S. are carefully followed at medical health care clinics. This data underscore the need for regular dental care. As PHIV youth were at higher risk for cavities, it will be important to better understand this relationship in order to develop targeted interventions.

## Introduction

Dental caries/decay and gingivitis remain the most common chronic infectious diseases in children in the US. It is estimated that 40–90% of children are affected by this “silent epidemic”. [1, 2] These rates seem abysmal since systemic and topical fluoride, sealants, and other preventative strategies are available for caries and gingivitis prevention and endorsed widely. [3–5] Disadvantaged minority children, who include many HIV-infected children, are at highest risk for poor dental health.[1] Lack of access to dental health care despite access to medical care [6, 7], poor nutrition, and frequent consumption of snacks are all contributing factors. The role of HIV-associated immunosuppression, however, is not well defined. HIV-infected children may also be at additional risk for caries due to their prolonged exposure to sugar-containing medications and potential for HIV-associated salivary gland dysfunction. [6]

Results from dental assessments in HIV-infected children vary widely with an 85% prevalence of caries reported in developing countries, [7, 8] while in a US study, only 20% of HIV-infected children ages 3–15 years had caries. [9] Furthermore, 7% to 20% of HIV infected children in developing countries were found to have gingivitis and no studies to date have reported the prevalence of periodontitis among HIV-infected children and adolescents in the US. [10, 11] In contrast to caries and periodontal disease, oral mucosal manifestations of HIV have dramatically decreased in children primarily due to antiretroviral therapy. (6) Because perinatally HIV-infected (PHIV) children in the US are aging, and adolescence is associated with an increasing prevalence of caries and periodontal disease, it is critical to assess the magnitude of oral disease in this population.

This study is one of the first to investigate the prevalence of oral mucosal, dental and periodontal disease through a comprehensive oral examination in a large, multi-site cohort of PHIV youth as compared to perinatally HIV-exposed but uninfected (PHEU) youth living in similar environments.

## Materials and Methods

### Study Design and Population

We conducted a cross-sectional study within the Adolescent Master Protocol (AMP) of the Pediatric HIV/AIDS Cohort Study (PHACS). AMP is a prospective cohort study designed to determine the impact of HIV infection and antiretroviral therapy (ART) on PHIV youth. AMP includes a comparison group of perinatally HIV-exposed but uninfected (PHEU) youth. AMP eligibility criteria included perinatal HIV infection or exposure and age 7 to < 16 years at enrollment. [12, 13] Regularly scheduled visits included audio-computer assisted structured interviews (ACASI), physical examination, and chart reviews for medication, diagnoses, CD4 counts and viral load (VL). [12, 13] Participants enrolled in AMP from March 2007 through October 2009 at 15 US clinical research sites. For this Oral Health protocol, sites were required to have either an affiliated dental school or dentist. Participants were enrolled from September 2012 through January 2014. Exclusion criteria included anesthesia being required to complete a dental examination, craniofacial anomalies that prevented completion of a comprehensive oral examination and a history of head or neck radiation. All eligible subjects from participating sites were asked to enroll.

Institutional Review Boards (IRB) at clinical sites and the Harvard T.H. Chan School of Public Health approved the study. Parents/legal guardians provided written informed consent for their child’s participation. Youth consented/assented per local IRB guidelines.

### Variables and Measures

#### Data Collection Overview

Sites scheduled the Oral Health study visit within 3 months of the regularly scheduled AMP annual visit. All attempts were made to keep the dentist blinded to HIV diagnosis, but this was not possible in all cases. A detailed oral health history was obtained which included the frequency of brushing, flossing, snacks/meals and juice/soda intake, perceived oral health, oral symptoms such as dry mouth, pain, difficulty swallowing, and utilization of dental care. A 5-minute unstimulated whole saliva flow rate was then performed. [14]

#### Training and Calibration of oral health examinations

Dentists at each site received training on standardized oral mucosal, dental, and periodontal examinations, measurement of plaque and gingival inflammation indices, and collection of oral specimens. Most sites had only one dentist, two sites had two and one had three. Each dentist examiner received a training module consisting of a presentation of clinical oral mucosal lesion slides made by one of the authors (CHS), followed by a Question & Answer session. After viewing the training module the dentists completed a calibration exercise that required them to review 40 clinical slides/pictures that illustrate oral disease outcomes, and to determine the correct clinical diagnoses for conditions depicted in the slides/pictures. Examiners had to correctly diagnose the conditions shown in 80% of the slides/pictures presented to be considered calibrated on the oral mucosal examination. None failed the calibration exercises.

Training and calibration with respect to dental and periodontal measures were administered to each site dentist by one of the authors (MR) as part of a site visit to each clinical research site prior to the study opening for accrual. Standardized techniques to measure caries, plaque index, gingival index, periodontal probing, bleeding on probing, and gingival margin levels were reviewed. [15, 16] To demonstrate standardized procedures and calibrate examiners, three pediatric patients were available for examination during each on-site visit. After appropriate consent, standardized techniques were demonstrated by MR on a patient including scoring for caries, plaque index, gingival index, bleeding on probing, probing depths and gingival margin to cemento-enamel junction. Following the demonstration, both the site dentist(s) and MR conducted the standardized examination on one of the two remaining pediatric patients. Successful agreement between the two examiners was defined by achieving a 90% concordance with respect to probing depths and gingival margin to cemento-enamel junction ± 1mm for all examined sites on a half mouth of one of the pediatric patients. If this level of agreement was not met, discrepancies were discussed between MR and the dentist. The calibration exercises were repeated on the half mouth of the second pediatric patient. If this 90% concordance level was not met, clinical parameters would be reviewed again and a calibration exercise conducted on a third pediatric patient. For all 16 examiners at all 11 sites, additional examined pediatric patients were not required to meet this 90% concordance level for probing depths and gingival margin position. The mean (SD, range) concordance level was 97.7% (2.4%, 92% - 100%) for probing depths and 98.3% (2.4%, 92% - 100%) for gingival margin.

For caries calibration, an examination was performed on a half mouth of the second pediatric patient. The surfaces of each tooth in the half mouth (occlusal, distal, mesial, buccal and lingual) were scored for the presence or absence of a clinically detectable carious lesion by the calibration examiner (MR) followed by the same examination by the site examiner(s). A minimum 90% concurrence for all examined surfaces was required for successful calibration as described for probing depths and gingival margin. The mean (SD, range) concordance level among the 16 examiners was 99.2% (1.3%, 96% -100%).

#### Oral Mucosal Examination End Points

Oral HIV/AIDS Research Alliance case definitions were used for oral mucosal disease with the inclusion of mucosal disease seen commonly in children. [17, 18]

#### Dental Disease and Periodontal Parameter End Points

The decayed-missing-filled-surfaces index (DMFS for permanent teeth and decayed-extracted-filled surfaces (defs) index for deciduous dentition with the M and e component of the index defined as missing or extracted due to caries) was used to assess the prevalence of dental caries experience. [19] While the DMFS reflects both past and current dental disease, the number of decayed surfaces (DS), a subset of the DMFS score, reflects active disease. The DMFS and DS also reflect severity of dental disease since it considers all surfaces of each tooth. DMFS or DS scores range from 0 to 128 (*i*.*e*., 4 surfaces examined for each of 12 incisal and canine teeth, and 5 surfaces for the other 16 permanent teeth). We additionally derived the decayed-missing-filled-teeth index (DMFT and deft) and the number of decayed (untreated) teeth for both permanent and deciduous teeth (DT and dt, respectively)—both reflect the extent of disease. DMFT reflects active and past dental disease whereas DT and dt reflect active disease. As for the DMFS, the M and e component of the index are defined as missing or extracted due to caries. DMFT and DT range from 0 to 28. The vast majority of participants (91%) had no deciduous teeth—therefore we combined permanent and deciduous teeth. Some participants did not have all 28 teeth scored mostly due to un-eruption or orthodontic work. To adjust for possible bias that might be caused by different number of present teeth, we multiplied the actual tooth count of DMFT or DT by the ratio of 28 over the number of scored teeth, with the assumption that the risk of caries for the absent teeth is the same as the present teeth for a given participant. All analyses on DMFT and DT were performed on the adjusted counts.

One buccal and one lingual site of all teeth were examined for plaques and the Plaque Index (PI: percentage of teeth with visible plaque) was evaluated as an objective measure of oral hygiene. [15] Probing depths and position of the gingival margin on 6 sites per tooth were assessed for periodontal clinical attachment loss (CAL). [20] Bleeding on probing (BOP) was also recorded on 6 sites per tooth on all teeth.

Based on the periodontal parameters CAL and probing depth, periodontitis was defined as either absent, mild, moderate, or severe. [21] Among those with no periodontitis, gingivitis was defined as having BOP on at least 10% of the sites scored. [22] All patients were referred for further dental care if needed.

### Statistical Analysis

The DMFS, DS, DMFT and DT scores were calculated for each participant and summarized by HIV status. PHIV participants were further grouped according to either CD4+ nadir (>200, ≤200 cells/mm^3^), current CD4+ counts (>200, ≤200 cells/mm^3^) or VL (<400, ≥400 copies/mL). As better measures of extent of disease, DMFT and DT of each of these PHIV sub-groups were compared to PHEU youth for crude association using a Wilcoxon test.

Periodontal disease was composed of either periodontitis or gingivitis. Periodontal disease (none, gingivitis, mild periodontitis, moderate periodontitis, or severe periodontitis) was compared between PHIV subgroups and PHEU group using Fisher’s Exact tests; the proportions of participants with any type of mucosal disease were compared between PHIV and PHEU youth by Fisher’s Exact test.

Distributions of DMFT and DT scores were tested for an excess proportion of zero counts and over-dispersion when compared to the Poisson distribution by likelihood ratio tests. As the tests were significant, zero-inflated negative binomial regression (ZINB) models were applied to assess the effects of HIV infection and other covariates on DMFT and DT scores. Univariable ZINB models for DMFT and DT score were first fit for HIV infection status, age, sex, race, ethnicity, caregiver characteristics, sexual history, substance use, oral health history and habits. Multivariable models were then constructed to contain HIV infection status, demographics, and other covariates with p-value ≤ 0.2 from univariable models. Covariates were selected separately for the zero-inflation portion and the negative-binomial portion of the model. The overall effect of each covariate on the mean score estimated from a ZINB model was derived by combining the effects from the two portions and reporting the overall mean ratio with a 95% confidence interval and p-value. [23, 24]

Ordinal logistic regression models were applied to assess whether PHIV versus PHEU youth differed in their odds of having periodontal disease. The assumption of proportional odds was examined using a Chi-square test. Multivariable models were constructed including all covariates with p-value ≤ 0.2 from univariable models without further variable selection.

Among PHIV youth, we evaluated associations of oral health outcomes with current VL and CD4+ count, CD4+ nadir and history of AIDS-defining illness, adjusting for covariates identified in the above multivariable models. Use of liquid medications was not examined since very few PHIV youth (5% or less) currently used them.

## Results

### Participant Characteristics and Oral Health History

Eleven clinical research sites enrolled 335 participants (209/ 376 PHIV youth, and 126/ 204 PHEU youth active in AMP by September 2012). Enrolled participants were similar to those not enrolled with respect to age, sex, race, ethnicity, and HIV infection status (data not shown). Demographics and laboratory studies are described in Table 1. Their distributions are consistent with what has been observed in the overall AMP cohort.[12, 25]

**Table 1:** Socio-demographic and health characteristics at oral health exam by HIV status among participants in the Pediatric HIV/AIDS Cohort Study (PHACS) Oral Health Substudy.

Characteristic	PHIV^f^ (n = 209) n (%)	PHEU^g^ (n = 126) n (%)	P-Value*
**Female**	110 (53)	64 (51)	0.82
**Race/ethnicity**			0.02
**Non-Hispanic black/African-American**	132 (63)	63 (50)	
**Non-Hispanic white**	9 (4)	8 (6)	
**Hispanic**	56 (27)	52 (41)	
**Other**	12 (6)	3 (2)	
**Age (years)**			< .001
**10–13**	34 (16)	55 (44)	
**14–16**	64 (31)	49 (39)	
**17–18**	63 (30)	13 (10)	
**19–22**	48 (23)	9 (7)	
**Maximum Tanner stage**			0.002
**1–3**	28 (14)	35 (28)	
**4**	38 (18)	30 (24)	
**5**	143 (68)	61 (48)	
**Sexual history**^a^			
**Ever had sex**^#^	99 (47)	40 (32)	0.004
**Ever had oral sex**	86 (41)	34 (27)	0.009
**Smoked cigarettes in past 3 months**^b^	22 (11)	7 (6)	0.16
**Used marijuana in past 3 months**^a^	49 (23)	14 (11)	0.006
**Drank alcohol in past 3 months**^a^	52 (25)	21 (17)	0.08
**Caregiver is biological parent**	79 (38)	102 (81)	< .001
**Caregiver is high school graduate**^c^	146 (70)	87 (69)	0.90
**Caregiver income <$20,001 annually**^d^	88 (42)	81 (64)	< .001
**Nadir CD4 cell count (cells/mm**^**3**^**)**			
**< 200**	70 (33)		
**200–350**	50 (24)		
**> 350**	89 (43)		
**Current CD4 cell count (cells/mm**^**3**^**)**			
**< 200**	14 (7)		
**200–350**	25 (12)		
**> 350**	170 (81)		
**Current HIV RNA load (copies/mL)**^e^			
**< 400**	142 (68)		
**≥ 400**	65 (31)		
**History of an AIDS-defining illness**	51 (24)		

* Fisher’s Exact test

^#^Included oral, vaginal and anal sex.

^a^7 missing

^b^9 missing

^c^3 missing

^d^8 missing

^e^2 missing

^f^perinatally HIV-infected

^g^perinatally HIV-exposed but uninfected

Only half of youth brushed their teeth twice a day, and 84% did not floss daily. Three-quarters had a regular source for dental care. Most youth reported frequent meals, snacking and sweet beverages. There were no differences in dental hygiene behaviors or food/beverage intake frequencies by HIV status (Table 2).

**Table 2:** Dietary habits, oral health history, and oral hygiene by HIV status among participants in the Pediatric HIV/AIDS Cohort Study (PHACS) Oral Health sub-study.

Characteristic	PHIV (n = 209) n (%)	PHEU (n = 126) n (%)	P-Value*
**Meal or snack intake frequency**			0.75
**1–3 per day**	25 (12)	17 (13)	
**4 per day**	43 (21)	22 (17)	
**5 or more per day**	141 (67)	87 (69)	
**Juice or soda intake frequency**			0.91
**0–3 per day**	125 (60)	78 (62)	
**4 per day**	35 (17)	19 (15)	
**5 or more per day**	49 (23)	29 (23)	
**Frequency of tooth brushing**			0.65
**Twice per day or more**	104 (50)	67 (53)	
**Once per day**	86 (41)	51 (40)	
**Never/occasionally**	19 (9)	8 (6)	
**Frequency of flossing**			0.14
**Twice per day or more**	13 (6)	6 (5)	
**Once per day**	17 (8)	19 (15)	
**Never/occasionally**	179 (86)	101 (80)	
**Have source of dental care**^a^	165 (79)	94 (75)	0.68
**Dental cleaning in past year**^b^	133 (64)	78 (62)	0.73
**Saliva flow rate (mL/min)**^c^ **median(min, max)**	0.70 (0.02, 3.00)	0.60 (0.00, 2.20)	0.09
**Percent teeth with visible plaque**			0.19
**<10%**	46 (22)	26 (21)	
**10 to <30%**	72 (34)	33 (26)	
**30% or more**	91 (44)	67 (53)	

*Wilcoxon test for saliva volume, Fisher’s Exact test for all the other characteristics

^a^5 missing

^b^1 missing

^c^2 missing

### Dental symptoms and disease

Oral symptoms were common, with 29% reporting tooth pain in the past year and 39% reporting that their gums bled occasionally when brushing their teeth. No differences were seen by HIV status regarding dryness, pain or bleeding. PHIV youth, however, reported more sores in their mouth than PHEU youth [38 (18%) vs. 13 (10%); p = 0.04].

Sixty-one percent of PHIV and 50% of PHEU youth had at least one tooth with caries (p = 0.04). PHIV youth had a greater mean DT than PHEU youth [2.2 (SD 3.4) vs 1.4 (SD 2.1); p = 0.04]. There was a similar trend for DS for PHIV vs. PHEU youth [3.1 (SD 6.5) vs 1.8 (SD 2.9); p = 0.06]. No crude association was found between HIV infection status and mean DMFT score [5.2 (SD 4.9) vs 5.0 (SD 4.7); p = 0.76] nor DMFS score [8.6 (SD 11.4) vs. 7.5 (SD 7.9); p = 0.90]. Overall, 47% had gingivitis, 16% had mild, and 16% had moderate periodontitis (none had severe).

Compared to PHEU youth, mean DT and DMFT scores were higher in PHIV youth with a CD4 nadir ≤200 cells/mm^3^, but similar to those with a nadir > 200 cells/mm^3^. Findings were similar for current CD4+ count comparisons and for current VL comparisons on mean DT score, but there were no differences in DMFT score by VL (Table 3). Presence of and severity of periodontal disease also did not differ by any HIV disease marker (Table 3).

**Table 3:** Oral diseases by nadir CD4 cell count, current CD4 cell count, or viral load.

By nadir CD4 cell count						
Characteristic		PHEU	PHIV		PHIV	
		(N = 126)	Nadir CD4 ≥200 (N = 139)	P-Value*	Nadir CD4 <200 (N = 70)	P-Value*
**DT**^a^ **score**	**median (min, max)**	0.50 (0, 11)	1 (0, 11)	0.27	1 (0, 19)	0.005
	**mean (sd)**	1.42 (2.08)	1.73 (2.39)		3.20 (4.59)	
**DMFT**^b^ **score**	**median (min, max)**	4 (0, 20)	4 (0, 26)	0.45	6 (0, 21)	0.05
	**mean (sd)**	5.00 (4.71)	4.50 (4.41)		6.61 (5.59)	
**Periodontal disease**^c^	**none**	25 (20%)	31 (23%)	0.70	11 (16%)	0.52
	**gingivitis**	65 (52%)	60 (44%)		32 (47%)	
	**mild periodontitis**	17 (13%)	22 (16%)		16 (24%)	
	**moderate periodontitis**	19 (15%)	24 (18%)		9 (13%)	

By current CD4 cell count						
Characteristic		(N = 126)	Current CD4 ≥200 (N = 195)	P-Value*	Current CD4 <200 (N = 14)	P-Value*
**DT**^a^ **score**	**median (min, max)**	0.50 (0, 11)	1 (0, 19)	0.11	2.50 (0, 19)	0.002
	**mean (sd)**	1.42 (2.08)	1.99 (2.93)		5.50 (6.28)	
**DMFT**^b^ **score**	**median (min, max)**	4 (0, 20)	4 (0, 26)	0.96	9 (0, 20)	0.04
	**mean (sd)**	5.00 (4.71)	4.93 (4.66)		9.07 (6.87)	
**Periodontal disease**^c^	**none**	25 (20%)	38 (20%)	0.44	4 (29%)	0.29
	**gingivitis**	65 (52%)	85 (45%)		7 (50%)	
	**mild periodontitis**	17 (13%)	35 (18%)		3 (21%)	
	**moderate periodontitis**	19 (15%)	33 (17%)		0 (0%)	

By viral load						
Characteristic		(N = 126)	VL <400 (N = 142)	P-Value*	VL ≥400 (N = 65)	P-Value*
**DT**^a^ **score**	**median(min, max)**	0.50 (0, 11)	1 (0, 19)	0.16	1 (0, 19)	0.01
	**mean (sd)**	1.42 (2.08)	1.87 (2.74)		3.08 (4.33)	
**DMFT**^b^ **score**	**median(min, max)**	4 (0, 20)	4 (0, 21)	0.72	4 (0, 21)	0.25
	**mean (sd)**	5.00 (4.71)	4.71 (4.41)		5.98 (5.30)	
**Periodontal disease**^c^	**none**	25 (20%)	30 (22%)	0.53	12 (19%)	1.00
	**gingivitis**	65 (52%)	59 (42%)		33 (52%)	
	**mild periodontitis**	17 (13%)	26 (19%)		11 (17%)	
	**moderate periodontitis**	19 (15%)	24 (17%)		8 (13%)	

* PHIV subgroup compared to PHEU. Wilcoxon test for DT and DMFT scores, Fisher’s Exact test for periodontal disease

^a^DT = number of decayed teeth

^b^DMFT = decayed-missing-filled teeth score

^c^4 missing information on periodontal disease

Oral mucosal disease was uncommon among the PHIV youth with ≤ 1% having each of the following: candidiasis (n = 3), hairy leukoplakia (2), recurrent HSV (1), recurrent aphthous ulcer (2), linear gingival erythema (2), and parotid enlargement (2). However, the combined prevalence was significantly higher in PHIV youth than PHEU youth (6% versus 1%; p = 0.02).

### Regression Analyses

#### DMFT score

DMFT scores did not differ by HIV status either with or without adjustment for other covariates. In univariable models (S1 Table), higher DMFT scores were observed in participants who were older, Tanner stage 5, had lower caregiver income, ever had sex, ever had oral sex, drank alcohol, smoked cigarettes, used marijuana, had no source of dental care, had at least 4 juice/sodas a day, and had low salivary flow rate. PHIV youth with a nadir and current CD4 count <200 cells/mm3 also had higher DMFT scores.

In the multivariable model (Table 4), higher DMFT scores were observed in participants who were older, had a biological parent as their caregiver, had a high frequency of juice/soda (≥5), and a low salivary flow rate (mL/min) (overall mean ratios shown in Table 4; S2 Table shows the full multivariable ZINB model). Among PHIV youth, DMFT scores no longer differed by CD4 count or VL after adjustment for similar covariates (see S3 Table).

**Table 4:** Zero-inflated negative binomial multivariable model of decayed-missing-filled teeth (DMFT) score (N = 311).

Parameter	Adjusted Mean Ratio* (95% CI)	P-Value
**PHIV youth**	0.97 (0.78, 1.19)	0.76
**Age (vs <14 years)**		
**14–16 years**	1.31 (0.97, 1.76)	0.08
**17–18 years**	1.73 (1.16, 2.60)	0.008
**19–22 years**	1.89 (1.24, 2.91)	0.003
**Female**	1.18 (0.97, 1.44)	0.10
**Black (vs non-black)**	0.84 (0.63, 1.13)	0.25
**Hispanic (vs non-Hispanic)**	0.93 (0.68, 1.26)	0.64
**Tanner stage (vs 1–3)**		
**Stage 4**	0.90 (0.64, 1.29)	0.58
**Stage 5**	0.80 (0.55, 1.18)	0.27
**Caregiver is biological parent**	1.16 (1.01, 1.32)	0.03
**Caregiver income <$20,001 annually**	1.10 (0.89, 1.36)	0.37
**Reported ever having sex**	0.99 (0.76, 1.29)	0.95
**Drank alcohol in past 3 months**	1.21 (0.90, 1.61)	0.20
**Smoked cigarettes in past 3 months**	1.04 (0.75, 1.42)	0.82
**Used marijuana in past 3 months**	1.16 (0.85, 1.58)	0.36
**Brushed teeth (vs ≥2 times/day)**		
**<1 time/day**	0.90 (0.72, 1.12)	0.36
**1 time/day**	1.04 (0.96, 1.13)	0.32
**Have no regular source of dental care**	1.13 (0.90, 1.40)	0.29
**Juice or soda (vs 0–3 times/day)**		
**4 times/day**	1.20 (0.93, 1.56)	0.16
**≥5 times/day**	1.39 (1.12, 1.73)	0.003
**Saliva flow rate (mL/min)**	0.78 (0.63, 0.96)	0.02

*Overall adjusted mean ratio estimate was evaluated by combining the estimates from the zero-inflated portion and the negative-binomial portion. Separate estimates are presented in S2 Table.

#### Number of decayed teeth (DT)

In the univariable analysis, higher DT scores were observed in participants who were PHIV, older, had a caregiver with less than a high school education, had low caregiver income, used marijuana in the past 3 months, had no source of dental care, had no teeth cleaning within the last year, had at least 5 meals/snacks per day, and had at least 4 juice/sodas per day. Among PHIV youth, those with a nadir and current CD4+ cell count <200 cells/mm3 and high VL (≥400) had higher DT scores (S4 Table).

In the multivariable model (Table 5), PHIV youth were more likely to have higher DT scores. Those with no source of regular dental care and a high frequency of meals/snacks (≥5 /day) and juice/soda (≥5 /day) were also more likely to have high DT scores. Female sex, low caregiver income and high PI had marginal significance. Among PHIV youth, DT scores no longer differed by current/nadir CD4 cell counts or VL after adjustments for similar covariates (S3 Table).

**Table 5:** Zero-inflated negative binomial multivariable model of number of decayed teeth (DT) (N = 312).

Parameter	Adjusted Mean Ratio* (95% CI)	P-Value
**PHIV youth**	1.69 (1.16, 2.46)	0.006
**Age (vs <14 years)**		
**14–16 years**	1.30 (0.81, 2.09)	0.28
**17–18 years**	1.69 (0.89, 3.24)	0.11
**19–22 years**	1.11 (0.52, 2.37)	0.79
**Female**	1.40 (1.00, 1.94)	0.05
**Black (vs non-black)**	0.88 (0.53, 1.44)	0.61
**Hispanic (vs non-Hispanic)**	1.01 (0.61, 1.67)	0.99
**Tanner Stage (vs 1–3)**		
**Stage 4**	0.98 (0.56, 1.72)	0.95
**Stage 5**	0.63 (0.35, 1.13)	0.12
**Caregiver is high school graduate**	0.80 (0.57, 1.12)	0.19
**Caregiver income < $20,001 annually**	1.41 (1.00, 1.97)	0.05
**Drank alcohol in past 3 months**	1.12 (0.64, 1.96)	0.70
**Smoked cigarettes in past 3 months**	1.40 (0.81, 2.42)	0.23
**Used marijuana in past 3 months**	1.20 (0.68, 2.11)	0.53
**Have no regular source of dental care**	2.26 (1.50, 3.40)	< .001
**Did not have teeth cleaned in past year**	1.12 (0.79, 1.60)	0.52
**Meal or snack (vs 1–3 times/day)**		
**4 times/day**	1.20 (0.65, 2.23)	0.56
**≥5 times/day**	1.86 (1.13, 3.08)	0.02
**Juice or soda (vs 0–3 times/day)**		
**4 times/day**	1.23 (0.78, 1.92)	0.38
**≥5 times/day**	1.62 (1.11, 2.35)	0.01
**Percent teeth with visible plaque**	1.01 (1.00, 1.01)	0.05

*Overall adjusted mean ratio was the same as the mean ratio from the negative binomial portion since the zero-inflated portion had intercept only.

#### Periodontal Disease

In univariable analysis (S5 Table), the odds of periodontal disease increased with age [Odds Ratio (OR) for 19 years and older vs. 14 years and younger = 2.17 (95% CI 1.15, 4.09)]. The odds were also higher among those who were Tanner stage 5 (vs. 3 or less) [OR = 2.01 (1.17, 3.46)], reported ever sexual activity [OR = 1.73; (1.14, 2.63)] and with high PI [OR = 1.02 per unit (1.01, 1.03)]. In multivariable analysis, the odds of periodontal disease only increased with PI, with 2% higher odds for every one additional percent of teeth with visible plaque [adjusted OR = 1.02 (1.01, 1.03); p < 0.001].

## Discussion

This is the first comprehensive study of dental, periodontal, and oral mucosal disease outcomes among PHIV and PHEU children and adolescents in the U.S. Despite frequent contact with the medical and dental care systems, the prevalence of poor oral health—both dental caries and periodontal disease—was high. We found a high rate of past and present dental disease as measured by DMFT and DMFS scores, with 61% having at least one untreated caries and 80% with any periodontal disease.

The prevalence of untreated caries among PHIV youth in our study is 4 times higher than the Healthy People 2010 target for untreated caries among adolescents of 15%. [26] This finding is of concern, given the overall decline in caries in permanent teeth between 1988–94 and 1999–2004 reported in the general adolescent population. [27] In the National Health and Nutrition Examination Survey (NHANES) the prevalence of untreated tooth decay among 16–19 year olds declined from 24% in 1988–1994 to 19% in 2011–2012. [28] The mean DMFT in this age group declined from 4.12 (SE 0.16) in 1988–94 to 3.25 (0.11) in 1999–2002, compared to a mean DMFT of 6.15 (SE 0.49) in our study population in the same age group (data not shown in the result section). High DMFT scores reflecting past disease as well as current among the PHIV may be explained in part by the past use of multiple medications in sugar-containing liquid formulations which have been shown to be cariogenic by some investigators. [29]

Dental caries is a multifactorial, transmissible disease that involves dissolution of mineralized tooth structure by acids produced by dental plaque bacteria. [30–32] The high number of untreated decayed teeth found among PHIV youth, in particular those with nadir or current CD4 ≤ 200 cells/mm^3^, is not well explained. The loss of association with CD4 counts in the multivariate sub-group analysis suggests this finding may be confounded by other factors not studied in this analysis.

Periodontal disease is an umbrella diagnosis which includes gingivitis and periodontitis. Eighty percent of our PHIV youth had some form of periodontal disease, with about half having gingivitis and a third demonstrating mild or moderate periodontitis. Similar to caries, CD4 count and VL did not influence the presence of periodontal disease with adjustment of other factors among PHIV youth. Although the Healthy People 2010 Objectives do not have a target for adolescents, it set a target of 41% for gingivitis among adults aged 35 to 44 years—a rate half of what was found with gingivitis or periodontitis in our population. [26] The high rate of gingivitis has potential implications for HIV disease progression. Gingivitis has been linked with systemic immune activation which in turn is associated with HIV disease progression. [33, 34]

Despite the high prevalence of dental and periodontal disease, oral mucosal disease was almost non-existent, which may reflect successful ART. The discrepancy between the high frequency of caries and low frequency of oral mucosal disease remains poorly understood. However, it is possible that dental caries are more affected by oral microbiomes which are altered in the face of HIV or ART.

In our analysis, PHIV youth were more likely to have a higher number of decayed teeth, independent of other factors. DMFT scores or periodontal disease, however, did not differ by HIV status. Other factors that influenced caries were expected including a high frequency of meals and sweet beverages, lack of a regular source of dental care and low salivary volume. [35] Our chosen comparison group was a population of youth who were perinatally exposed to HIV. With regards to socioeconomic status which is known to influence oral health, this group provided an appropriate comparison group. [36] However, these uninfected youth were also exposed to the oral microbiomes of their HIV infected mothers which is known to strongly influence the child’s oral microbiome and thereby the child’s oral health. [37] This in part may have explained the lack of consistent differences observed by HIV status for all the oral health parameters. On the other hand, similarities in socioeconomic status may also explain the lack of differences.

No other published studies comparing oral health in PHIV and PHEU youth were available for comparison. A Bronx study of 102 PHIV children aged 3–15 years reported 23% had untreated caries and 21% had gingivitis. [9] However, these children were much younger than our youth with a mean age of 7.8 years. Similarly, studies in sub-Saharan Africa and in Brazil enrolled younger children than ours. One study of Kenyan HIV-infected children reported 20% of 12–15 year-olds had untreated caries. [38] In Uganda, 10% of 237 HIV-infected children aged 1.5 to 12 years had untreated caries in their permanent teeth with 20% having gingivitis.[11] Much more similar to our rates was a study in 120 West African children. This study reported a past or present caries prevalence of 86% and the mean DMFT score was 4.9 (SD 4). [8] Another study in Mozambique reported that HIV-infected children receiving ART had higher rates of caries than those not on ART suggesting that the liquid ART medications may contribute to caries. [39]

The primary limitation of this study is the cross sectional design making causality difficult to assess. In addition, the use of the DMFT and DMFS indices, although standards of research, have their own limitations since these indices weigh caries, missing and restored teeth similarly. We are planning a longitudinal follow up of this population as well as the examination of other co-factors such as the oral microbiome.

## Conclusion

In conclusion, oral health was poor in our PHIV youth, despite frequent interactions with the medical system. The rate of untreated caries seems inexcusably high since most caries are preventable with fluoride and sealants. Health care providers caring for these youth should monitor their dental health histories with annual referral to dentists. The high rate of gingivitis observed is worrisome since in adults, periodontitis has been associated with systemic immune activation and disease progression. Studies of systemic immune activation and their potential association with periodontal disease are underway in this cohort. Although the relationship with HIV status requires further study, it is clear that further work is needed to target appropriate interventions for preventing caries and gingivitis in HIV-infected youth.

## Supporting Information

S1 Table Univariable zero-inflated negative binomial models of decayed-missing-filled-teeth (DMFT) score. (PDF)

S2 Table Multivariable zero-inflated negative binomial model of decayed-missing-filled-teeth (DMFT) score. (PDF)

S3 Table Multivariable zero-inflated negative binomial models of decayed-missing-filled-teeth (DMFT) and decayed teeth (DT) among HIV positive participants, adjusted for covariates in the core multivariable models. (PDF)

S4 Table Univariable zero-inflated negative binomial models of number of decayed teeth (DT). (PDF)

S5 Table Univariable ordinal logistic regression models of periodontal disease. (PDF)
