# Supplementary material for: The Burden of Oral Disease among Perinatally HIV-Infected and HIV-Exposed Uninfected Youth
Source: PLoS One. 2016 Jun 14;11(6):e0156459. doi: 10.1371/journal.pone.0156459 (PMC4907464; doi:10.1371/journal.pone.0156459)
Supplement: S1 Table — (PDF) [file pone.0156459.s001.pdf]

Supplemental Table 1.

Univariable zero-inflated negative binomial models of decayed-missing-filled-teeth (DMFT) score.

Parameter	N	Overall Mean Ratio* (95% CI)	P-Value
PHIV infection	335	1.04 (0.84, 1.29)	0.70
Age (vs <14 years)	335		
14-16 years		1.29 (0.99, 1.67)	0.06
17-18 years		1.64 (1.24, 2.17)	0.001
≥19 years		2.05 (1.54, 2.73)	<0.001
Female	335	1.16 (0.94, 1.43)	0.16
Black (vs non-black)	335	0.85 (0.68, 1.05)	0.12
Hispanic (vs non-Hispanic)	335	1.17 (0.94, 1.45)	0.15
Tanner stage (vs 1-3)	335		
Stage 4		1.10 (0.79, 1.54)	0.56
Stage 5		1.54 (1.14, 2.06)	0.004
Caregiver is biological parent	335	1.17 (0.95, 1.45)	0.13
Caregiver is high school graduate	332	0.91 (0.73, 1.14)	0.42
Caregiver income <$20,001 annually	327	1.26 (1.02, 1.55)	0.03
Reported ever having sex	328	1.49 (1.22, 1.82)	<0.001
Reported ever having oral sex	328	1.40 (1.14, 1.71)	0.001
Drank alcohol in past 3 months	328	1.65 (1.32, 2.07)	<0.001

Smoked cigarettes in past 3 months	326	1.41 (1.02, 1.95)	0.04
Used marijuana in past 3 months	328	1.67 (1.34, 2.08)	<0.001
Brushed teeth (vs ≥2 times/day)	335		
<1 time/day		0.84 (0.53, 1.31)	0.44
1 time/day		0.97 (0.79, 1.20)	0.80
Flossed teeth (vs ≥2 times/day)	335		
<1 time/day		0.92 (0.60, 1.43)	0.72
1 time/day		0.80 (0.43, 1.50)	0.49
Have no regular source of dental care	330	1.26 (0.99, 1.59)	0.06
Did not have teeth cleaned in past year	334	1.01 (0.82, 1.25)	0.94
Meal or snack (vs 1-3 times/day)	335		
4 times/day		0.88 (0.61, 1.25)	0.47
≥5 times/day		0.97 (0.71, 1.34)	0.87
Juice or soda (vs 0-3 times/day)	335		
4 times/day		1.38 (1.06, 1.79)	0.02
≥5 times/day		1.57 (1.25, 1.97)	<0.001
Saliva flow rate (mL/min)	333	0.80 (0.64, 0.98)	0.03
Percent teeth with visible plaque	335	1.00 (0.99, 1.01)	0.75
Nadir CD4 cell count (vs >350 cells/mm^3)	209		
<200 cells/mm^3		1.72 (1.29, 2.28)	<0.001
200-350 cells/mm^3		1.48 (1.08, 2.02)	0.01

Current CD4 cell count (vs >350 cells/mm^3)	209		
<200 cells/mm^3		1.88 (1.22, 2.89)	0.004
200-350 cells/mm^3		1.18 (0.80, 1.73)	0.41
Current HIV RNA load ≥400 copies/mL (vs <400)	207	1.27 (0.97, 1.66)	0.08
History of an AIDS-defining illness	209	1.16 (0.88, 1.52)	0.29

*Overall adjusted mean ratio estimate was evaluated by combining the estimates from the zero-inflated portion and the negative-binomial portion of the ZINB model.
