# Supplementary material for: The Burden of Oral Disease among Perinatally HIV-Infected and HIV-Exposed Uninfected Youth
Source: PLoS One. 2016 Jun 14;11(6):e0156459. doi: 10.1371/journal.pone.0156459 (PMC4907464; doi:10.1371/journal.pone.0156459)
Supplement: S2 Table — (PDF) [file pone.0156459.s002.pdf]

Supplemental Table 2.

Multivariable zero-inflated negative binomial model of decayed-missing-filled-teeth (DMFT) score.

Parameter	Odds of Zero Inflation		Adjusted Count Ratio	
	aOR (95% CI)	P-Value	Ratio Estimate (95%CI)	P-Value
Intercept	1.89 (0.68, 5.23)	0.22	5.78 (3.95, 8.46)	<.001
PHIV infection			0.97 (0.78, 1.19)	0.76
Age (vs <14 years)				
14-16 years	0.53 (0.18, 1.59)	0.26	1.24 (0.92, 1.66)	0.16
17-18 years	0.60 (0.13, 2.85)	0.52	1.66 (1.12, 2.48)	0.01
≥19 years	0.15 (0.02, 1.49)	0.11	1.69 (1.10, 2.58)	0.02
Female	0.63 (0.27, 1.52)	0.31	1.13 (0.94, 1.37)	0.21
Black (vs non-black)			0.84 (0.63, 1.13)	0.25
Hispanic (vs non-Hispanic)			0.93 (0.68, 1.26)	0.64
Tanner stage (vs 1-3)				
Stage 4	0.44 (0.11, 1.73)	0.24	0.85 (0.59, 1.21)	0.37
Stage 5	0.57 (0.15, 2.21)	0.42	0.76 (0.52, 1.10)	0.15
Caregiver is biological parent	0.26 (0.11, 0.64)	0.003		
Caregiver income <$20,001 annually	0.81 (0.33, 1.97)	0.64	1.08 (0.88, 1.32)	0.47

Parameter	Odds of Zero Inflation		Adjusted Count Ratio	
	aOR (95% CI)	P-Value	Ratio Estimate (95%CI)	P-Value
Reported ever having sex	1.23 (0.44, 3.46)	0.70	1.01 (0.78, 1.31)	0.93
Drank alcohol in past 3 months			1.21 (0.90, 1.61)	0.20
Smoked cigarettes in past 3 months			1.04 (0.76, 1.42)	0.82
Used marijuana in past 3 months	0.15 (0.01, 1.85)	0.14	1.03 (0.76, 1.40)	0.86
Brushed teeth (vs ≥2 times/day)				
<1 time/day	2.21 (0.64, 7.60)	0.21		
1 time/day	0.65 (0.26, 1.61)	0.35		
Have no regular source of dental care			1.13 (0.91, 1.40)	0.29
Juice or soda (vs 0-3 times/day)				
4 times/day	0.60 (0.15, 2.37)	0.47	1.16 (0.90, 1.49)	0.26
≥5 times/day	0.47 (0.16, 1.40)	0.18	1.31 (1.06, 1.62)	0.01
Saliva flow rate (mL/min)			0.78 (0.63, 0.96)	0.02
