# Supplementary material for: The Burden of Oral Disease among Perinatally HIV-Infected and HIV-Exposed Uninfected Youth
Source: PLoS One. 2016 Jun 14;11(6):e0156459. doi: 10.1371/journal.pone.0156459 (PMC4907464; doi:10.1371/journal.pone.0156459)
Supplement: S3 Table — (PDF) [file pone.0156459.s003.pdf]

Supplemental Table 3.

Multivariable zero-inflated negative binomial models of decayed-missing-filled-teeth (DMFT) and decayed teeth (DT) among HIV positive participants, adjusted for covariates in the core multivariable models.

Parameter	DMFT[a]		DT[b]	
	Overall aMR* (95% CI)	P-Value	Overall aMR* (95% CI)	P-Value
Nadir CD4 cell count (vs >350 cells/mm^3)				
<200 cells/mm^3	1.20 (0.90, 1.60)	0.20	1.01 (0.61, 1.69)	0.96
200-350 cells/mm^3	1.24 (0.94, 1.64)	0.13	1.23 (0.75, 2.03)	0.41
Current CD4 cell count (vs >350 cells/mm^3)				
<200 cells/mm^3	1.21 (0.77, 1.89)	0.41	1.29 (0.59, 2.83)	0.53
200-350 cells/mm^3	1.01 (0.70, 1.45)	0.97	1.21 (0.63, 2.32)	0.56
Current HIV RNA load ≥400 copies/mL (vs <400)	0.88 (0.67, 1.14)	0.33	1.03 (0.63, 1.68)	0.91
History of an AIDS-defining illness[c]	0.96 (0.74, 1.23)	0.73	1.34 (0.83, 2.17)	0.23

* Overall adjusted mean ratios were the same as the mean ratios from the negative binomial portions since the variables displayed were only included in the negative binomial portion.

[a] Adjusted for all covariates listed in Table 4.

[b] Adjusted for all covariates listed in Table 5.

[c] History of an AIDS-defining illness indicates if ever CDC Class C.
