# Supplementary material for: The Burden of Oral Disease among Perinatally HIV-Infected and HIV-Exposed Uninfected Youth
Source: PLoS One. 2016 Jun 14;11(6):e0156459. doi: 10.1371/journal.pone.0156459 (PMC4907464; doi:10.1371/journal.pone.0156459)
Supplement: S4 Table — (PDF) [file pone.0156459.s004.pdf]

Supplemental Table 4.

Univariable zero-inflated negative binomial models of number of decayed teeth (DT).

Parameter	N	aMR* (95% CI)	P-Value
PHIV infection	335	1.57 (1.12, 2.20)	0.009
Age (vs <14 years)	335		
14-16 years		1.26 (0.82, 1.93)	0.30
17-18 years		1.76 (1.11, 2.79)	0.02
≥19 years		1.78 (1.08, 2.92)	0.02
Female	335	1.28 (0.93, 1.77)	0.14
Black (vs non-black)	335	0.95 (0.67, 1.33)	0.75
Hispanic (vs non-Hispanic)	335	1.16 (0.82, 1.64)	0.40
Tanner stage (vs 1-3)	335		
Stage 4		1.21 (0.71, 2.05)	0.48
Stage 5		1.38 (0.89, 2.14)	0.15
Caregiver is biological parent	335	0.92 (0.67, 1.28)	0.63
Caregiver is high school graduate	332	0.60 (0.42, 0.85)	0.004
Caregiver income <$20,001 annually	327	1.43 (1.03, 1.98)	0.03
Reported ever having sex	328	1.20 (0.86, 1.67)	0.28
Reported ever having oral sex	328	1.18 (0.84, 1.65)	0.34
Drank alcohol in past 3 months	328	1.37 (0.93, 2.00)	0.11
Smoked cigarettes in past 3 months	326	1.51 (0.87, 2.64)	0.14
Used marijuana in past 3 months	328	1.50 (1.01, 2.25)	0.05

Brushed teeth (vs ≥2 times/day)	335		
<1 time/day		0.85 (0.46, 1.57)	0.60
1 time/day		0.82 (0.59, 1.16)	0.27
Flossed teeth <1 time/day (vs ≥1)	335	1.18 (0.76, 1.84)	0.47
Have no regular source of dental care	330	2.28 (1.58, 3.30)	<.001
Did not have teeth cleaned in past year	334	1.78 (1.28, 2.47)	<.001
Meal or snack (vs 1-3 times/day)	335		
4 times/day		1.32 (0.71, 2.46)	0.38
≥5 times/day		2.14 (1.26, 3.62)	0.005
Juice or soda (vs 0-3 times/day)	335		
4 times/day		1.77 (1.15, 2.75)	0.01
≥5 times/day		1.74 (1.19, 2.55)	0.005
Saliva flow rate (mL/min)	333	1.00 (0.69, 1.44)	0.99
Percent teeth with visible plaque	335	1.01 (1.00, 1.01)	0.10
Nadir CD4 cell count (vs >350 cells/mm^3)	209		
<200 cells/mm^3		2.17 (1.39, 3.40)	<.001
200-350 cells/mm^3		1.50 (0.90, 2.47)	0.12
Current CD4 cell count (vs >350 cells/mm^3)	209		
<200 cells/mm^3		3.01 (1.46, 6.19)	0.003
200-350 cells/mm^3		1.68 (0.94, 3.00)	0.08
Current HIV RNA load ≥400 copies/mL (vs <400)	207	1.65 (1.09, 2.50)	0.02

History of an AIDS-defining illness	209	1.42 (0.90, 2.24)	0.13

*Overall mean ratio was the same as the mean ratio from the negative binomial portion since the zero-inflated portion had intercept only for all characteristics except for saliva flow rate, for which the overall mean ratio estimate was evaluated by combining the estimates from the zero-inflated and negative binomial portion
