# Supplementary material for: The Burden of Oral Disease among Perinatally HIV-Infected and HIV-Exposed Uninfected Youth
Source: PLoS One. 2016 Jun 14;11(6):e0156459. doi: 10.1371/journal.pone.0156459 (PMC4907464; doi:10.1371/journal.pone.0156459)
Supplement: S5 Table — (PDF) [file pone.0156459.s005.pdf]

Supplemental Table 5.

Univariable ordinal logistic regression models of periodontal disease.

Parameter	N	OR* (95% CI)	P-Value
PHIV infection	331	1.17 (0.77, 1.77)	0.47
Age (vs <14 years)	331		
14-16 years		1.41 (0.83, 2.38)	0.20
17-18 years		1.81 (1.01, 3.26)	0.05
≥19 years		2.17 (1.15, 4.09)	0.02
Female	331	0.73 (0.49, 1.10)	0.13
Black (vs non-black)	331	1.00 (0.66, 1.53)	0.99
Hispanic (vs non-Hispanic)	331	0.97 (0.63, 1.49)	0.88
Tanner stage (vs 1-3)	331		
Stage 4		1.14 (0.59, 2.18)	0.70
Stage 5		2.01 (1.17, 3.46)	0.01
Caregiver is biological parent	331	0.87 (0.58, 1.30)	0.49
Caregiver is high school graduate	328	1.18 (0.76, 1.85)	0.46
Caregiver income <$20,001 annually	323	0.87 (0.58, 1.31)	0.49
Reported ever having sex	325	1.73 (1.14, 2.63)	0.01
Reported ever having oral sex	325	1.36 (0.89, 2.09)	0.16
Drank alcohol in past 3 months	325	1.35 (0.83, 2.21)	0.23
Smoked cigarettes in past 3 months	323	1.55 (0.75, 3.18)	0.24
Used marijuana in past 3 months	325	1.30 (0.78, 2.18)	0.32

Brushed teeth (vs ≥2 times/day)	331		
<1 time/day		1.10 (0.51, 2.37)	0.80
1 time/day		1.05 (0.69, 1.61)	0.82
Flossed teeth (vs ≥2 times/day)	331		
<1 time/day		1.14 (0.47, 2.72)	0.78
1 time/day		1.68 (0.59, 4.81)	0.33
Have no regular source of dental care	327	1.00 (0.61, 1.64)	0.99
Did not have teeth cleaned in past year	330	0.84 (0.55, 1.28)	0.42
Meal or snack (vs 1-3 times/day)	331		
4 times/day		0.74 (0.35, 1.54)	0.42
≥5 times/day		0.83 (0.44, 1.55)	0.55
Juice or soda (vs 0-3 times/day)	331		
4 times/day		0.92 (0.52, 1.61)	0.76
≥5 times/day		1.30 (0.79, 2.14)	0.30
Saliva flow rate (mL/min)	329	1.44 (0.94, 2.21)	0.10
Percent teeth with visible plaque	331	1.02 (1.01, 1.03)	<.001
Nadir CD4 cell count (vs >350 cells/mm^3)	205		
<200 cells/mm^3		1.45 (0.80, 2.63)	0.22
200-350 cells/mm^3		1.46 (0.76, 2.81)	0.25
Current CD4 cell count (vs >350 cells/mm^3)	205		
<200 cells/mm^3		0.57 (0.21, 1.59)	0.28
200-350 cells/mm^3		1.19 (0.54, 2.66)	0.67
Current HIV RNA load ≥400 copies/mL (vs	203	0.90 (0.52, 1.56)	0.71

<400)			
History of an AIDS-defining illness	205	1.42 (0.78, 2.59)	0.25

*Proportional odds of disease.
